# Immune-associated biomarkers for early diagnosis of Parkinson’s disease based on hematological lncRNA–mRNA co-expression

**DOI:** 10.1042/BSR20202921

**Published:** 2020-12-21

**Authors:** Kecheng Lei, Liwen Zhang, Yijing He, Hui Sun, Weifang Tong, Yichun Xu, Lingjing Jin

**Affiliations:** 1 Neurotoxin Research Center of Key Laboratory of Spine and Spinal Cord Injury Repair and Regeneration of Ministry of Education, Neurological Department of Tongji Hospital, Tongji University School of Medicine, Shanghai 200065, P.R. China; 2 Department of Pathology and Laboratory Medicine, Emory University School of Medicine, Atlanta 30322, GA, U.S.A.; 3 National Engineering Research Center for Biochip, ShanghaiBiochip Limited Corporation, Shanghai 201203, P.R. China; 4 Department of Pathology, Shanghai Tongji Hospital, Tongji Hospital Affiliated to Tongji University, Shanghai 200065, P.R. China

**Keywords:** Biomarkers, Immune response, Integrative genomics, LncRNA-mRNA coexpression network, Parkinson's disease

## Abstract

Early stage diagnosis of Parkinson’s disease (PD) is challenging without significant motor symptoms. The identification of effective molecular biomarkers as a hematological indication of PD may help improve the diagnostic timelines and accuracy. In the present paper, we analyzed and compared the blood samples of PD and control (CTR) patients to identify the disease-related changes and determine the putative biomarkers for PD diagnosis. Based on the RNA sequencing analysis, differentially expressed genes (DEGs) were identified, and the co-expression network of DEGs was constructed using the weighted gene correlation network analysis (WGCNA). The analysis leads to the identification of 87 genes that were exclusively regulated in the PD group, whereas 66 genes were significantly increased and 21 genes were significantly decreased in contrast with the control group. The results indicate that the core lncRNA–mRNA co-expression network greatly changes the immune response in PD patients. Specifically, the results showed that Prader Willi Angelman Region RNA6 (PWAR6), LINC00861, AC83843.1, IRF family, IFIT family and calcium/calmodulin-dependent protein kinase IV (CaMK4) may play important roles in the immune system of PD. Based on the findings from the present study, future research aims at identifying novel therapeutic strategies for PD.

## Introduction

Parkinson’s disease (PD) is the second most common neurodegenerative disease in the world [1]. With the increase in age, the prevalence of the disease increases. Almost 2% of the population over the age of 60 is diagnosed with PD, and an average 3–5% of the population over the age of 85 have PD [2]. The diagnosis of early-stage PD is difficult, because motor symptoms are only observable in late-stage PD with over 50% of dopaminergic neurons loss in the substantia nigra [3]. On the other hand, diagnosis methods for PD using non-motor symptoms are either not fully developed or not widely accepted by the clinical practices due to the limited effectiveness and accessibility [4]. For example, although cerebrospinal fluid (CSF) is a precise measurand for the diagnosis of PD [5], the painful procedure, limited accessibility, and high cost may drive patients away from such diagnosis methods. While various researches have proposed alternative, easily accessible methods for the early-stage diagnosis of PD, effective biomarkers are yet to be identified.

Mounting researches have shown the correlation between PD and the immune system. The key feature of a healthy immune system is that it can correctly identify ‘self’ and ‘non-self’ when enhancing immune response [6]. A complete immune response contains cell-mediated immune response and humoral immune response [7]. Lymphocytes in circulating blood account for approximately 80–90% of T cells and can be distinguished by the differentiated antigen cell membrane, which binds to specific mAbs [8]. These cell antigens are assigned to a cluster of differentiation (CD) [6]. In 1985, the researchers observed that the signs of immunosuppression in PD patients were similar to those of normal aging, but the number of CD4^+^ T cells was reduced [9]. It was reported that the ratio of CD4^+^ to CD8^+^ T lymphocytes decreased due to the regulation of blood T lymphocytes in PD patients, suggesting that peripheral immune responses were also present in PD pathology [10]. Moreover, the ratio of CD95/CD3 increases in the lymphocytes of PD patients; however, after L-dopa treatment, it decreases significantly [11]. Thus, it is evident that the CD families plays a significant role in the pathology of PD. The identification of early biomarkers in immune cells has the potential to enable the currently available methods to quickly diagnose early-stage PD.

In addition to immune biomarkers, long non-coding RNAs (lncRNAs) are also demonstrated to have the potential to elucidate PD pathogenesis. LncRNAs are one of the non-coding RNAs with length greater than 200 nt that participate in various biological processes (BPs) such as stem cell differentiation, organ development, epigenetic regulation, and immune system regulation [12]. It has been indicated that lncRNA has crucial regulatory potential in protein transcription and post-transcriptional processing [13]. In addition, experimental evidence suggests that lncRNA can regulate brain evolution and intermediary behaviors in the central nervous system (CNS) [14]. In Alzheimer’s disease (AD), overexpression of BACE1 antisense RNA (BACE1-AS), which is an lncRNA, causes the deterioration of AD in the brain [15]. In Huntington’s disease, the nuclear paraspeckle assembly transcript 1 (lncRNA NEAT1) has been shown to promote neuroprotection by adding neurons [16]. Ultimately, it is evident that the expression of lncRNA plays an important role in the deterioration of neurodegenerative diseases such as PD. Therefore, identifying the differentially expressed lncRNA and its molecular mechanism has the potential to signal PD at an early stage.

To identify candidate biomarkers for early-stage PD diagnosis, novel sequencing and bioinformatics methods were adopted. Unlike the traditional test where only one gene can be sequenced at a time, whole transcriptome sequencing is a powerful tool that enables the analysis of thousands of genes in patients [17]. It rapidly scans for candidate biomarkers, which helps discover the underlying mechanism of PD [18]. In addition, weighted gene co-expression network analysis (WGCNA) [19], a bioinformatics analysis method, has been proven to effectively detect the complex module–trait relationships [20]. The distinct advantage for WGCNA is its capability to cluster genes into a model or network according to the weight correlation coefficient between genes, and then analyzes the correlation between modules and sample characteristics. These methods were adopted in this work to identify candidate biomarkers and analyze the correlations among the candidates.

As a summary, in the present study, we performed the whole transcriptome sequencing using blood samples from PD patients and healthy controls to analyze the differential expression profiles of lncRNAs and mRNAs. Subsequently, a co-expression network of lncRNA–mRNA module–trait relationships was constructed by WGCNA. We then performed gene ontology (GO) functional annotation, Kyoto Encyclopedia of Genes and Genomes (KEGG) pathway analysis, and protein–protein interaction (PPI) analysis. Finally, several differentially regulated proteins are identified, which are also correlated to the immune system and can be validated as the biomarkers of the lncRNA–mRNA network for early-stage PD diagnosis.

## Materials and methods

### Patients and ethics statement

The blood samples of age-matched PD patients and healthy control were diagnosed and collected at Shanghai Tongji Hospital (Table 1). The healthy control (without PD and any other systemic neurodegenerative diseases) were voluntary participants. All experiments were performed following the relevant guidelines and regulations at Tongji Hospital. The approval number of the present study by ethics committee is KYSB-2017-097. Written consent was obtained from all patients.

**Table 1:** The clinical information of patients

Sample	Sex	Age	Duration of symptoms (Y)	HY stages
Healthy people				
A	M	66	-	-
B	F	62	-	-
C	F	58	-	-
PD patient				
D	M	73	2	2
E	F	61	4	2
F	F	69	0.5	1

Abbreviation: HY, Hoehn and Yahr scale.

### RNA extraction

Total RNA was extracted using TRIzol reagent (Invitrogen, California, U.S.A.) according to the manufacturer’s instructions. The RNA concentration was determined by NanoDrop One spectrophotometer (Thermo) and RNA quality was evaluated with the Agilent 4200 Bioanalyzer.

### Whole transcriptome sequencing data analysis

RNA sequencing was performed on an Illumina platform and paired-end 150 bp raw reads were generated. Clean reads were obtained by removing the adaptor, low-quality raw reads. Using HISAT (v2.0.5) [21] with default parameters, paired-end clean reads were mapped and StringTie (v1.3.3b) [22] was adopted to predict novel transcripts. Subsequently, the novel genes were annotated through the Pfam database. For gene expression level analysis, the FPKM (Fragments Per Kilo-base per Million reads) [23] of mRNA and lncRNA were calculated.

### Screening of differentially expressed genes

Differentially expressed genes (DEGs) were determined from PD patients and healthy controls through comparison of the normalized read count value using the R package (http://www.bioconductor.org/packages/release/bioc/html/edgeR.html), and the resulting *P*-values were adjusted using the Benjamini and Hochberg’s approach for controlling the false discovery rate (FDR). DEGs with statistical significance were identified through volcano plot filtering. Absolute log2 fold-change > 1 and *P*-value <0.01 were set as the cut-off criteria. Hierarchical clustering was performed using the pheatmap package in R [24].

### Construction of core regulatory network

All the normalized value of expressed genes were used to construct gene co-expression networks by applying the WGCNA package in R [20]. With default parameters (unsigned-type topological overlap matrix (TOM), power β was set to 20. The co-expression methodology was typically used to explore the correlation between gene expression levels. Genes involved in the same pathway or functional compound tend to show similar expression patterns. Therefore, the construction of a co-expression network facilitates the identification of genes with similar biological functions. The Venn Diagram package in R was used for overlapping between the pheatmap genes in a highly similar expression model and the DEGs. Finally, the core networks were visualized and analyzed using Cytoscape [25], and the genes with top connective degrees were defined as central hubs. The functional protein association network was analyzed by STRING software [26].

### GO and KEGG enrichment analysis

To understand the potential biological functions, we used Cluster Profiler on R platform (https://bioconductor.org/packages/release/bioc/html/clusterProfiler.html) and Metascape database platform. GO or KEGG terms with corrected *P*-value less than 0.05 were considered significantly enriched by DEGs.

### Validation of the expression in DEGs in q-PCR

The total RNA of blood samples for PD patients and CTR were obtained. Reverse transcription was performed with ReverTra Ace qPCR RT Kit (Toyobo, Japan), and primers were designed and purchased from Sangon Biotech. Real-time PCR was performed with Power SYBR™ Green PCR Master Mix (Life Technologies, U.S.A.) by ABI 7500 Real-Time PCR System. The relative quantification of the target genes was calculated by the comparative cycle threshold (*C*_T_) method (2^−ΔΔ*C*_T_^). The primers’ information is listed in Supplementary Table S1. The expression of GAPDH was used as an endogenous control. All tests were performed in triplicate [27].

### Statistical analysis

All statistical analyses were performed with the software packages described above and standard settings unless otherwise indicated. Data are presented as mean ± standard deviation (SD). Statistical analysis was performed with GraphPad Prism (GraphPad Software, U.S.A.). Statistical significance was assumed as *P*≤0.05.

## Results

### Expression profiles, GO terms, and KEGG pathways for differential transcriptome-wide expression between PD patients and healthy control in blood samples

**Figure 1:**
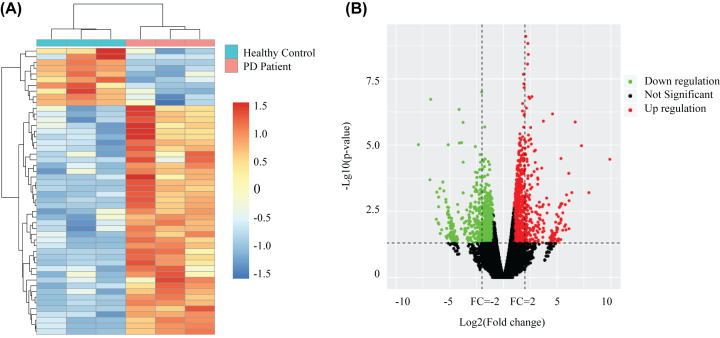
Expression profiles for differential transcriptome-wide expression between PD patients and healthy control in their blood samples (**A**) Hierarchical clustering shows a distinguishable transcriptome-wide expression profiling among groups. (**B**) Volcano analysis exhibit differentially transcriptome-wide expression. Red dots represent up-regulated genes. Green dots illustrate down-regulated genes.

The differential gene expression between PD patients and healthy control blood samples were examined. Hoehn and Yahr scale (HY) stage is a commonly used system for describing how the symptoms of PD progress. Stage 1 and 2 belong to the early stage of PD [28]. The expression profiles were derived based on RNA-sequencing analysis. These aberrant genes were presented as an expression heatmap (Figure 1A). A total of 716 up-regulated and 648 down-regulated genes were identified to be significant (*P*-value <0.05 and fold-change ≥ 2) (Figure 1B). These DEGs were therefore considered as potential key regulators to screen for the hub genes network in PD.

**Figure 2:**
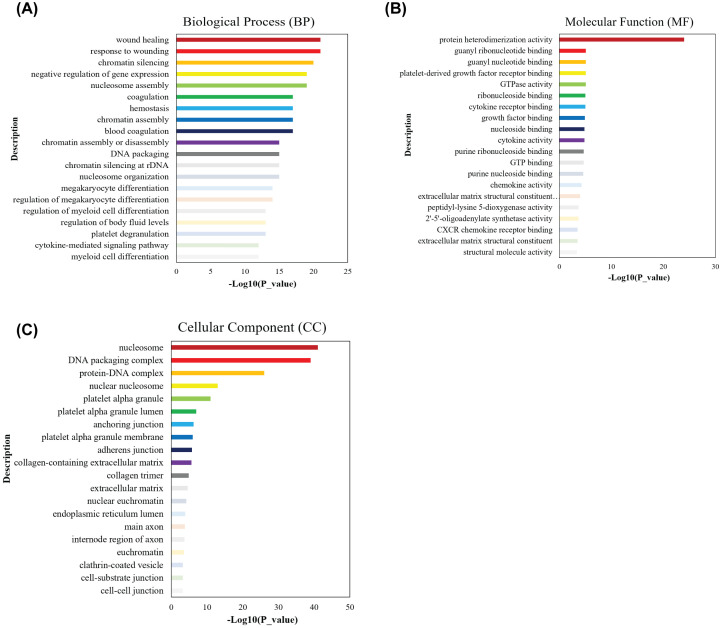
GO terms for differential transcriptome-wide expression between PD patients and healthy control in their blood samples (**A**) BPs, (**B**) MFs, and (**C**) CCs of GO terms.

In order to further understand the functions of these DEGs, we performed GO and KEGG enrichment analyses using the R package ‘cluster profile’ and Metascape database platform. GO terms included molecular functions (MFs), BPs, and cellular components (CCs). The MF was primarily associated with protein heterodimerization activity, the BP was primarily associated with wound healing, while the CC was enriched with nucleosome (Figure 2).

### WGCNA analysis using the lncRNA–mRNA co-expression network

**Figure 3:**
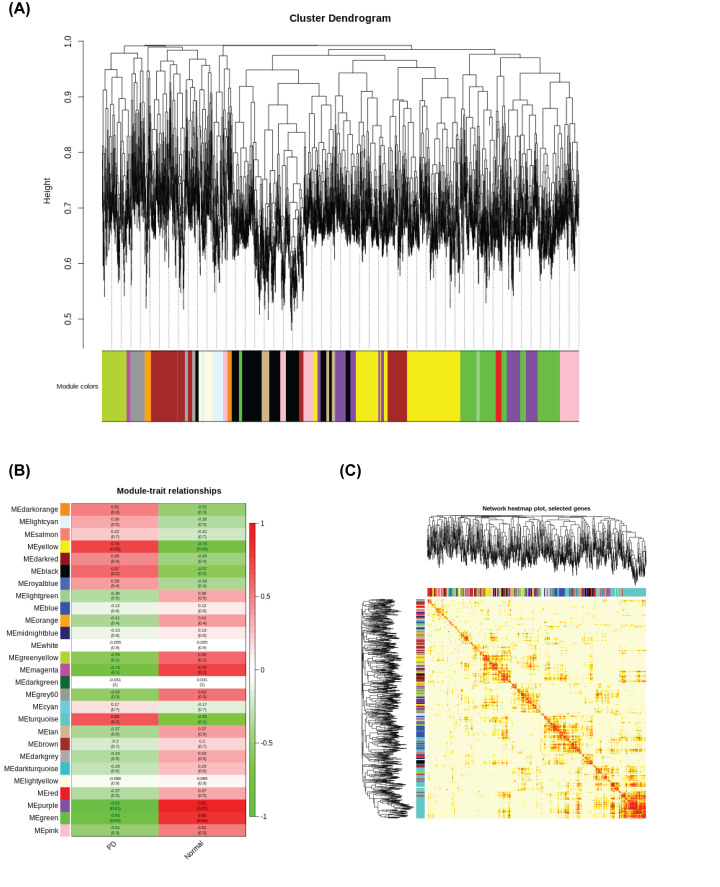
WGCNA analysis using the co-expression network (**A**) Clustering dendrograms of genes with dissimilarity based on the topological overlap, together with assigned module color. (**B**) Module–trait associations. Each row represents a module eigengene, each cell contains the corresponding correlation and *P*-value. (**C**) Heatmap plot of topological overlap in the gene network. In the heatmap, each row and column correspond to a gene, light color denotes low topological overlap, and progressively darker red denotes higher topological overlap. Darker squares along the diagonal correspond to modules. The gene dendrogram and module assignment are shown along the left and top.

All the normalized values of expressed genes were used to perform the co-expression network using the WGCNA package in R. After highly similar modules were merged, a total of 27 modules were identified in the co-expression network (Figure 3A). These co-expression modules were constructed and depicted in different colors. A total of 165 genes in the gray 60 modules did not belong to other modules, accounting for 1.1% of all total genes. The number of genes included in these modules was 777 (black module), 1524 (blue module), 1255 (brown module), 233 (cyan module), 88 (dark green module), 79 (dark gray module), 53 (dark orange module), 93 (dark red module), 82 (dark turquoise), 804 (green module), 490 (green-yellow module), 165 (gray 60 module), 208 (light cyan module), 165 (light green module), 124 (light yellow module), 596 (magenta module), 220 (midnight blue module), 73 (orange module), 743 (pink module), 554 (purple module), 798 (red module), 123 (royal blue module), 435 (salmon module), 466 (tan module), 3195 (turquoise module), 49 (white module), and 970 (yellow module). The module–trait relationships were constructed by the WGCNA algorithm. Modules with higher Spearman’s correlation coefficients are also the most important factors of modules associated with the trait. From Figure 3B, the modules classification was observed, including the two categories of ‘PD Patient’ and ‘Healthy Control.’ Among these modules, the purple-colored module was the most relevant for PD. This module includes the most relevant lncRNA expression profiles for PD. We further analyzed the interaction among 27 co-expression modules. In total, 1000 genes were selected at random for the heatmap (Figure 3C).

### The interacting proteins and GO functional enrichment analyses for genes analysis

**Figure 4:**
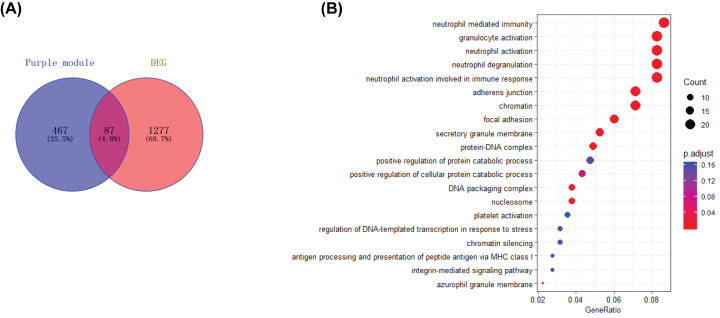
Functional annotation of DEGs in blood samples from patients with PD (**A**) A Venn diagram of the genes from the purple module and edgeR, generated using an online tool. Each colored circle represents a different dataset, and areas of overlap indicate shared genes. (**B**) Top 20 significant KEGG pathways. The −log10 (*P*-value) of each term is colored according to the legend.

As shown in Figure 4A, each dataset was initially analyzed separately to identify overlapping genes. A total of 1364 DEGs were identified in edgeR and 554 phenotype-specific genes in the purple-colored module constructed by WGCNA. There are 87 genes overlapping in the two datasets, suggesting that these related genes may be conservative. Among these 87 genes, 66 were up-regulated while only 21 were down-regulated. We further investigated the potential function of genes in PD by using GO analysis. The results with the genes revealed that they were enriched in a wide variety of immune-related functions, including neutrophil-mediated immunity, granulocyte activation, and neutrophil activation (Figure 4B). These data suggested that immune-related processes serve a significant role in PD.

**Figure 5:**
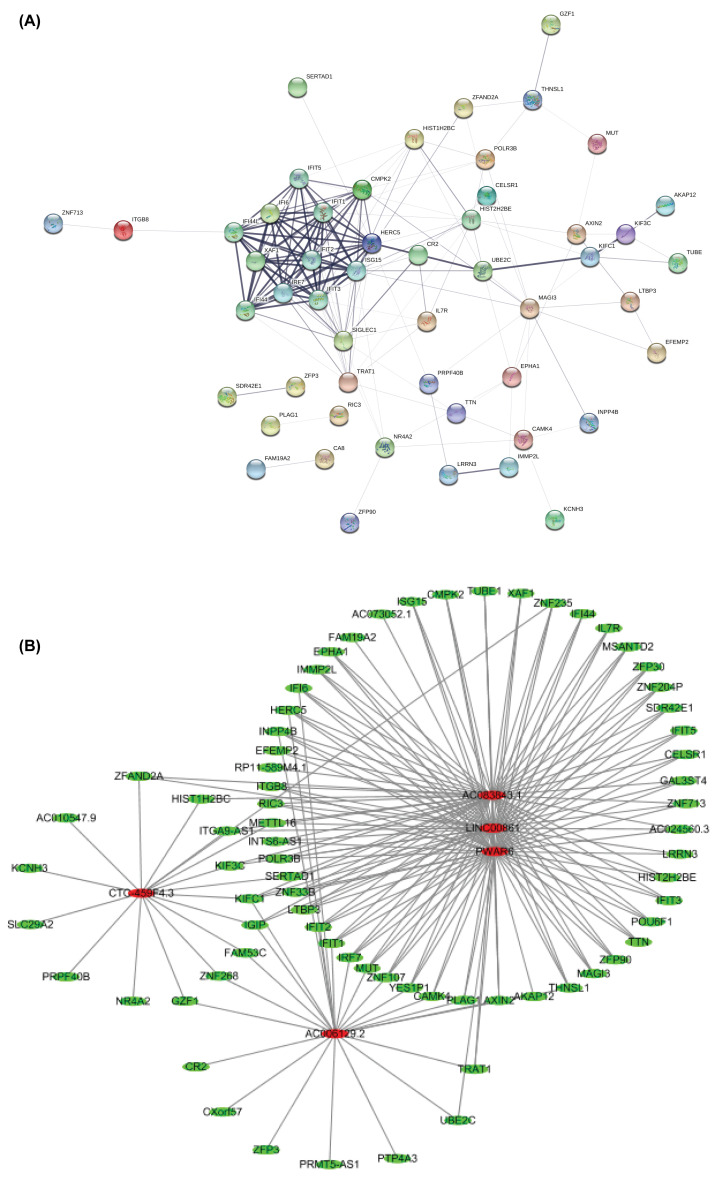
Functional annotations between gene and their potential drought-responsive targets (**A**) PPI of DEGs. The STRING protein database was utilized to analyze PPIs. (**B**) The global view of the lncRNA–mRNA network in PD.

To better understand which of these shared genes were most likely to be the key genes for PD, the STRING is used to predict PPIs, with which a PPI network for these 87 common genes was built (Figure 5A). These genes were deemed to be the hub genes for PD. Interestingly, IFIT1, IFIT2, IFIT3, IFIT, and IRF7 were in the core position of PPI networks. Again, the IFIT family and the IRF family are closely related to the immune response and are considered to be the key potential targets that are most likely to have effective activity.

Moreover, the most significant lncRNA module was further analyzed. In the lncRNA module (Figure 5B), Cytoscape was constructed to visualize the molecular interaction networks, integrate with gene expression profiles of databases and search in large networks. In the results, CTC-459F4.3, AC006129.2, AC083843.1, LINC00861, and Prader Willi Angelman Region RNA6 (PWAR6) regulated multiple co-expressed genes. Network analysis shows that genes can be jointly regulated by different lncRNAs, and one lncRNA can regulate multiple genes. The co-expressed genes may be the predicted target of lncRNA [29].

### Validation of the expression levels in candidate genes

**Figure 6:**
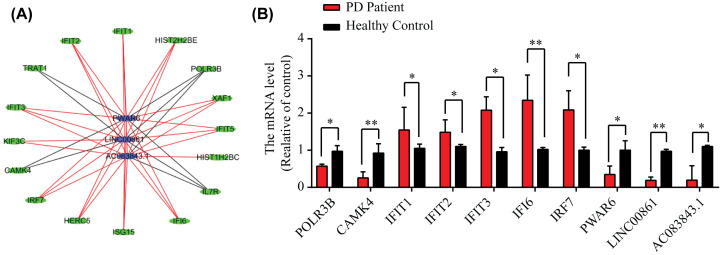
Expression profiles for differentially expressed mRNAs between PD patients and healthy control blood samples (**A**) The subnetwork of lncRNA and mRNA genes was related to the immune microenvironment. (**B**) The mRNA level of the selective genes by q-PCR. Data are means ± SEM (*P<0.05; **P<0.01, Student’s *t* test, *n*=3).

From functional enrichment analysis, the subnetwork of lncRNA and mRNA genes was related to the immune microenvironment (Figure 6A). Based on the results, we randomly selected 11 genes of the hub gene for qPCR validation, and these genes included IFIT1, IFIT2, IFIT3, IFIT5, IFI6, IRF7, ISG15, POLR3B, KIF3C, CAMK4, PWAR6, LINC00861, and AC83843.1. According to the qPCR confirmation, all the selected genes were regulated in PD which coincides with our integrated analysis (Figure 6B).

## Discussion

Nowadays, the relationship between immunity and PD is increasingly appreciated and attracting widespread attention [30–32]. To diagnose PD early and accurately, it is important to develop new diagnostic biomarkers. In this work, genes related to the immune microenvironment that are subsequently related to PD patients were identified. In particular, 87 genes involved in the immune response were extracted by comparing the overall gene expression of a large number of cases with high vs. low immune scores. As indicated in the result, PWAR6, LINC00861, AC83843.1, IRF family, IFIT family, and calcium/calmodulin-dependent protein kinase IV (CaMK4) played important roles in the immune system and may become a therapeutic target for PD.

Since PD is a neurodegenerative disease, several features in the brain and peripheral blood support the role of the immune system in PD [33]. The number of activated microglia in the blood and the infiltration of T cells in the CNS indicate that the PD process involves not only the local immune system but also the peripheral immune system [33]. For example, CD163^+^ macrophages also express antigen recognition and presentation molecules, which can transmit brain immune responses to the surrounding immune system [34]. Moreover, COX-2 can induce DA oxidation and NFκB-induced inflammation of microglia [35]. In addition, it induces the production of prostaglandins, which triggers the surrounding immune system [36]. In conclusion, microglia-derived factors and T cells serve an important role in the recruitment of peripheral immune cells, which consequently influences PD.

Through basic genetic and molecular research, many previous works have examined the complex molecular and cellular characteristics of PD, and various new genes have been identified which are involved in PD [37,38]. Due to the rapid breakthrough of whole genome sequencing technology, the clinical problems and related pathological mechanisms of various diseases have been studied and developed [39].

The IRFs can play a broader and more direct role in the regulation of inflammation and immunity because they control cell development and function, which participate in the immune response [40]. Since IRF1 and IRF2 are the earliest identified members of the IRF family, the researches started with these factors on IRF regulating immunity [41]. It is demonstrated that PINK1 promotes the VCAM-1 promoter by increasing the transcriptional activity of IRF-1in PD [42]. Meanwhile, the study used Irf1^−/−^ mouse embryo fibroblasts (MEFs) showed IRF-1 and p53 work together to deal with DNA damage [43]. It has also been demonstrated that KRAS-IRF 2 axis drives immunosuppression and immunotherapy resistance of colorectal cancer [44]. It was reported that IRF-7 is the main regulator of type I interferon-dependent immune response [45]. Moreover, IRF-7 is elevated in patients with advanced PD [46]. It was also reported that IRF-9 regulates the immune homeostasis of intestinal flora in mice [47]. Thus, the IRF family can induce homologous to the cellular immune response in PD. IRF-3 can directly regulate the IFIT gene, which is induced early in the immune response [48]. It is also indicated that IRF-8 attenuates the induction of interferon-induced by IFIT family members [49].

The IFIT family genes are also involved in many cellular and viral processes; meanwhile, they display conservative gene structure and gene arrangement [50]. Among the IFIT genes, IFIT1 suppresses lipopolysaccharide (LPS)-mediated Toll-like receptor 4 (TLR4) activation in mouse macrophages by showing silence, and also further suppresses induced downstream genes through unknown direct or indirect mechanisms [51]. It was reported that IFIT2 defined a new innate immune effector pathway against West Nile virus infection [52]. Herpes simplex virus 1 membrane protein UL41 can offset IFIT3 antiviral innate immunity [53]. Moreover, IFIT5 also enhanced the antiviral response by enhancing the innate immune signaling pathway [54]. As a summary, ITIF family limits replication and regulates adaptive immunity.

It was reported that differentially expressed mRNA and lncRNA are related to the development of PD. LncRNA HOTAIR via miR-126-5p promotes the progression of PD targets RAB3IP [55]. PWAR6, which is lncRNA with a processed exon of the *UBE3A-ATS/SNHG14* transcript [56], decreases the expression in PD patients. According to LncBase, PWAR6 is targeted by hsa-miR-125-5p and hsa-miR-30-5p. Meanwhile, CaMK4, which is also the target of hsa-miR-125-5p and hsa-miR-30-5p, decreased the expression in PD patient in our study. CaMK4 is a member of the CaMK family and has been shown to play an important role in immune responses including T-cell activation, development, and activation of various transcription factors [57]. It has been proven that CaMK4 is in dendritic cells derived from human monocytes (DC) through the TLR4 pathway [58]. *In vitro* experiments indicated that CaMK4 inhibition may serve as a therapeutic strategy for Th17-driven autoimmune diseases [59]. KN-93, which is a CaMK4 inhibitor, promoted the production and function of Foxp3^+^ regulatory T cells in MRL/*lpr* mice [60]. In PD, it was reported recently that DJ-1 regulated CaMKKβ/CaMK4/CREB1 activity to promote TH expression [61]. CaMK4, which is also included in the calcium signaling pathway, has shown abundant changes in the brain of an MPTP mouse model of PD [62]. In conclusion, PWAR6 may regulate miR-125-5p and CaMK4 in a ceRNA-dependent manner in PD.

There may be limitations to our study that should be noted: (1) this work is focused on the analysis of mRNA and lncRNA expression. Biochemical validation of the effects of deregulated ceRNA-like molecules and pathways will be an active area of future research when the appropriate tools are available. (2) The numbers of the patient samples are still not enough, further study should include more patient samples and PD datasets to increase the strength of the findings. (3) Further examination of dopaminergic neurons from the substantia nigra could support and refine the conclusions on disease etiology of different types of neurons both in the *in vitro* and *in vivo* settings.

## Conclusion

In summary, we identified the lncRNA–mRNA co-expression network associated with the immune microenvironment. These genes of the lncRNA–mRNA co-expression network have been validated in independent PD cohorts and may help outline the early biomarkers of PD patients. In addition, it will be interesting to test whether this new set of genes can provide a stronger predictor than a single gene. Finally, further research on these genes may provide comprehensive new insights into the potential correlation between the immune microenvironment and PD early biomarkers. We believe that our research results will lay the foundation for further meaningful research. Future studies will conduct in-depth research on the lncRNAs and mRNAs network to determine its function in the pathophysiology of PD.

## Supplementary Material

Supplementary Table S1

## Data Availability

The datasets generated and analyzed during the current study are not publicly available due to the ongoing study but are available from the corresponding author on reasonable request.

## Abbreviations

AD: Alzheimer’s disease
BACE1: beta-site amyloid precursor protein cleaving enzyme 1
BP: biological process
CaMK4: calcium/calmodulin-dependent protein kinase IV
CC: cellular component
CD: cluster of differentiation
ceRNA: competing endogenous RNA
CNS: central nervous system
COX-2: cyclooxygenase-2
DA: dopamine
DEG: differentially expressed gene
GO: gene ontology
HOTAIR: HOX transcript antisense RNA
IFIT: Interferon Induced proteins with Tetratricopeptide repeats
IRF: interferon regulatory factors
KEGG: Kyoto Encyclopedia of Genes and Genomes
lncRNA: long non-coding RNA
MF: molecular function
PD: Parkinson’s disease
PINK1: PTEN-induced kinase 1
PPI: protein–protein interaction
PWAR6: Prader Willi Angelman Region RNA6
TLR4: Toll-like receptor 4
VCAM-1: vascular cell adhesion protein 1
WGCNA: weighted gene co-expression network analysis
